# Statin intensity and postoperative mortality following open repair of intact abdominal aortic aneurysm

**DOI:** 10.1002/bjs5.94

**Published:** 2018-09-06

**Authors:** H. N. Alshaikh, F. Bohsali, F. Gani, B. Nejim, M. Malas

**Affiliations:** ^1^ Johns Hopkins Surgery Center for Outcomes Research Johns Hopkins School of Medicine Baltimore Maryland USA; ^2^ Department of Medicine Johns Hopkins Bayview Medical Center Baltimore Maryland USA; ^3^ Johns Hopkins Bayview Vascular and Endovascular Clinical Research Center Baltimore Maryland USA

## Abstract

**Background:**

There is a lack of evidence for the association between intensive statin therapy and outcomes following vascular surgery. The aim of this study was to evaluate the association between perioperative statin intensity and in‐hospital mortality following open abdominal aortic aneurysm (AAA) repair.

**Methods:**

Patients undergoing open AAA repair between 2009 and 2015 were identified from the Premier Healthcare Database. Statin use was classified into low, moderate and high intensity, based on American College of Cardiology/American Heart Association guidelines. Supratherapeutic intensity was defined as doses higher than the recommended guidelines. Multivariable logistic regression analyses were undertaken to assess the association between statin intensity and postoperative major adverse events and in‐hospital mortality.

**Results:**

Of 6497 patients undergoing open AAA repair, 3217 (49·5 per cent) received perioperative statin. Statin users were more likely to present with three or more co‐morbidities than non‐users (26·5 *versus* 21·8 per cent; *P* < 0·001). Unadjusted postoperative mortality was significantly lower in statin users (2·6 *versus* 6·3 per cent; *P* < 0·001); however, there was no difference in the risk of developing major adverse events. Multivariable analysis showed that statin use was associated with lower odds of death (odds ratio 0·41, 95 per cent c.i. 0·31 to 0·54). Moderate, high and supratherapeutic statin intensities were not associated with lower odds of death or major adverse events compared with low‐intensity statin therapy.

**Conclusion:**

Statin use is associated with lower odds of death in hospital following open AAA repair. High‐intensity statins were not associated with lower morbidity or mortality.

## Introduction

Morbidity and mortality rates following open repair of abdominal aortic aneurysm (AAA) remain high. Approximately 4 per cent of patients undergoing open AAA repair are not expected to survive the hospital stay^1^ ^2^, with 27–49 per cent developing postoperative complications^3^. Several pharmacological agents, including aspirin, anticoagulants, beta‐blockers and lipid‐lowering agents, have been suggested to reduce adverse events following open AAA repair^4^. Recently, statins have been shown to improve long‐term survival after AAA repair. In a meta‐analysis^5^ of patients who underwent either open or endovascular AAA repair, lipid‐modifying therapies were associated with a 39 per cent reduction in long‐term mortality. Among eight studies included in this meta‐analysis, five involved patients who underwent open AAA repair. However, two^6^ ^7^ of these were undertaken by a group of researchers who previously published multiple clinical trials of questionable scientific validity (an investigation by Erasmus University concluded that these trials were unreliable and contained fictitious data, and so the lead investigator was dismissed from the university)^6^, ^7^, ^8^. The other three retrospective studies had poor definitions of statin use^9^ ^10^ and/or did not perform analyses specific to patients undergoing open AAA repair^9^, ^10^, ^11^. In a more recent study of patients undergoing AAA repair^12^, the crude risk of in‐hospital, 30‐day, 90‐day and 1‐year postoperative death was similar in statin users and non‐users. This study also did not include an adjusted analysis for patients who underwent open repair.

Currently, the European Society of Cardiology and the European Society of Anaesthesiology (ESC/ESA)^13^ and American College of Cardiology/American Heart Association (ACC/AHA)^14^ guidelines recommend the initiation or continuation of statin therapy before non‐cardiac operations. However, evidence demonstrating the effect of perioperative statin therapy on outcomes following vascular surgery is currently lacking^15^. Neither the ESC/ESA nor the ACC/AHA guidelines provide specific recommendations on the optimum dose or type of statin to be used. In addition, the effect of statin intensity on outcomes following vascular surgery is unknown. The aim of the present study was to examine the association between statin intensity and in‐hospital morbidity and mortality following open AAA repair.

## Methods

The institutional review board at the Johns Hopkins School of Medicine approved the present study under exempt status. The Premier Healthcare Database (PHD) (Premier, Charlotte, North Carolina, USA) was used to identify the study cohort. The PHD is an all‐payer database that represents about 20 per cent of all annual inpatient discharges in the USA. It cumulatively maintains administrative, healthcare utilization and financial data from more than 700 hospitals. Hospital participation in this database is voluntary. At participating hospitals, a full data abstraction is performed for the included year. Premier acquires patient data from the health information management departments at participating hospitals through its Quality Advisor interface tool. To ensure accuracy of the data, Premier undertakes data validation at first data collection and at each subsequent monthly data submission. Furthermore, it reconciles the submitted data with the hospital financial statement and thresholds on cases, charges and cost before publishing these data in the PHD. When data errors are detected during this phase, Premier notifies the hospital and makes sure the data are correct in the source system. The corrected data are resubmitted to Premier, where the same data processing and validation guidelines are followed as in the original submission. A detailed description of the PHD is available elsewhere^16^.

### Setting and patient selection

Included patients were those who underwent open repair of intact (non‐ruptured) AAA between June 2009 and March 2015 in US hospitals affiliated with Premier. The ICD‐9‐CM diagnosis code 441·4 was used to identify admissions primarily for intact AAA, whereas ICD‐9‐CM procedure codes 38·34, 38·36, 38·44, 38·64, 39·25 and 39·52 were used to identify open AAA repairs. Exclusion criteria were: age less than 45 years, admission owing to trauma and multiple operations for AAA (defined as concurrent open and endovascular AAA repair during the same hospital admission). In addition, patients receiving a mean daily dose of statin larger than the 99th percentile were excluded from the final analysis.

### Study co‐variables

Data collected included: patient characteristics (age, sex, race, insurance status, admission type, co‐morbidities, complications and discharge status) and hospital characteristics (census region, and urban/rural and teaching status). Patient co‐morbidity was classified using the Charlson Co‐morbidity Index (CCI), with patients grouped into three co‐morbidity levels (CCI score 0–1, 2 and at least 3)^17^. The PHD differentiates ICD‐9‐CM diagnosis codes based on whether the patient was admitted with the code (a co‐morbidity), or acquired this code during admission (a complication). Major adverse events (MAE) were defined as the presence of any respiratory, cardiac, gastrointestinal, haemorrhagic, infectious, renal or neurological complications as defined by Shaw and colleagues^18^ (*Table S1*, supporting information). The ICD‐9‐CM diagnosis codes used to capture patient co‐morbidities are listed in *Table S2* (supporting information).

To capture in‐hospital use of beta‐blockers and statins, the PHD charge description master (CDM) file was queried. The CDM file contains a list of all bills incurred by a patient each day. These bills contain information on drugs, devices and supplies, medical procedures, diagnostic evaluations and other hospital services. Data on drugs include generic name, amount, unit, route of administration and number of times administered per day. Patients were classified as beta‐blocker or statin users if they received any dose of beta‐blocker or statin at any time during their hospital stay (perioperative). The in‐hospital mean daily dose of statin was calculated by dividing the total amount of statin received during the entire hospital stay by the total number of days it was administered. Statin use was further classified based on the ACC/AHA guideline^19^, which takes into account the type and daily dose of statin, into groups receiving low‐, moderate‐ and high‐intensity statins. Patients who received statin doses higher than the recommended guidelines were classified as having a supratherapeutic intensity of statin.

### Statistical analysis

The primary study outcome was in‐hospital postoperative mortality, and the main comparison was statin users *versus* non‐users. Secondary outcomes included all complications listed above and the composite MAE. Categorical variables were compared between study groups using Pearson's χ^2^ test or Fisher's exact test, as appropriate; continuous variables, presented as median (i.q.r.), were analysed using the Mann–Whitney *U* test. Multivariable logistic regression analyses were undertaken to assess the association between statin intensity and postoperative mortality and MAE. Variables known to have an effect on outcomes and those with *P* < 0·200 in univariable analysis were selected for inclusion in the multivariable analysis. Variables were further selected to achieve a parsimonious model based on the lowest Akaike information criterion values. *P* < 0·050 was considered statistically significant. Stata® MP version 14·1 (StataCorp, College Station, Texas, USA) was used for statistical analysis.

## Results

The range of statin dose by statin type and intensity is shown in *Table S3* (supporting information).

### Baseline characteristics of statin users *versus* non‐users

Some 6497 patients met the inclusion criteria, of whom 3217 (49·5 per cent) received statins in hospital (*Table* 1). Patients who received statins were slightly younger than those who did not. There was no difference in sex, race, insurance status, admission type or hospital location between statin users and non‐users. Similarly, there was no difference in smoking status, and history of end‐stage renal disease, chronic obstructive pulmonary disease (COPD) and arrhythmia between the two groups. Statin users were more likely to have a history of myocardial infarction (MI), coronary artery disease, congestive heart failure, hypertension and chronic kidney disease. Statin users were also more likely to have a CCI score of 3 or more, to receive perioperative beta‐blockers in hospital, and be treated in teaching hospitals.

**Table 1:** Baseline characteristics by statin use and statin intensity

	Whole cohort			Statin users				
	No statin	Statin		Low	Moderate	High	Supratherapeutic	
	(*n* = 3280)	(*n* = 3217)	*P* †	(*n* = 277)	(*n* = 1987)	(*n* = 586)	(*n* = 367)	*P* †
Age (years)*	71 (65–77)	70 (65–76)	0·043‡	72 (65–78)	70 (65–76)	69 (63–75)	70 (65–75)	0·001‡
Women	935 (28·5)	861 (26·8)	0·116	82 (29·6)	524 (26·4)	168 (28·7)	87 (23·7)	0·248
Non‐white	661 (20·2)	673 (20·9)	0·444	56 (20·2)	407 (20·5)	110 (18·8)	100 (27·2)	0·013
Insurance			0·887					0·223
Medicare	2365 (72·1)	2342 (72·8)		211 (76·2)	1445 (72·7)	421 (71·8)	265 (72·2)	
Medicaid	97 (3·0)	87 (2·7)		10 (3·6)	58 (2·9)	14 (2·4)	5 (1·4)	
Private	675 (20·6)	653 (20·3)		42 (15·2)	407 (20·5)	128 (21·8)	76 (20·7)	
Other	143 (4·4)	135 (4·2)		14 (5·1)	77 (3·9)	23 (3·9)	21 (5·7)	
CCI score			< 0·001					0·453
0–1	1582 (48·2)	1381 (42·9)		132 (47·7)	835 (42·0)	251 (42·8)	163 (44·4)	
2	984 (30·0)	983 (30·6)		77 (27·8)	631 (31·8)	173 (29·5)	102 (27·8)	
≥ 3	714 (21·8)	853 (26·5)		68 (24·5)	521 (26·2)	162 (27·6)	102 (27·8)	
Admission status			0·087					0·054
Elective	2620 (79·9)	2530 (78·6)		203 (73·3)	1561 (78·6)	480 (81·9)	286 (77·9)	
Urgent	266 (8·1)	311 (9·7)		38 (13·7)	196 (9·9)	47 (8·0)	30 (8·2)	
Emergency	394 (12·0)	376 (11·7)		36 (13·0)	230 (11·6)	59 (10·1)	51 (13·9)	
Beta‐blocker use	2534 (77·3)	2874 (89·3)	< 0·001	235 (84·8)	1779 (89·5)	527 (89·9)	333 (90·7)	0·074
Smoking			0·135					0·227
Never	1126 (34·3)	1115 (34·7)		94 (33·9)	693 (34·9)	198 (33·8)	130 (35·4)	
Ever	930 (28·4)	971 (30·2)		102 (36·8)	580 (29·2)	179 (30·5)	110 (30·0)	
Current	1224 (37·3)	1131 (35·2)		81 (29·2)	714 (35·9)	209 (35·7)	127 (34·6)	
History of MI	366 (11·2)	520 (16·2)	< 0·001	31 (11·2)	289 (14·5)	132 (22·5)	68 (18·5)	< 0·001
Coronary artery disease	1182 (36·0)	1677 (51·8)	< 0·001	120 (43·3)	985 (49·6)	366 (62·5)	206 (56·1)	< 0·001
Congestive heart failure	205 (6·3)	295 (9·2)	< 0·001	20 (7·2)	179 (9·0)	65 (11·1)	31 (8·4)	0·245
Hypertension	2470 (75·3)	2680 (83·3)	< 0·001	239 (86·3)	1636 (82·3)	491 (83·8)	314 (85·6)	0·208
Chronic kidney disease	472 (14·4)	585 (18·2)	< 0·001	51 (18·4)	358 (18·0)	103 (17·6)	73 (19·9)	0·823
End‐stage renal disease	24 (0·7)	28 (0·9)	0·531	2 (0·7)	20 (1·0)	5 (0·9)	1 (0·3)	0·568
COPD	1158 (35·3)	1176 (36·6)	0·293	106 (38·3)	707 (35·6)	226 (38·6)	137 (37·3)	0·516
Arrhythmia	422 (12·9)	438 (13·6)	0·373	34 (12·3)	266 (13·4)	91 (15·5)	47 (12·8)	0·468
Hospital type								
Urban	2963 (90·3)	2957 (91·9)	0·025	252 (91·0)	1831 (92·1)	542 (92·5)	332 (90·5)	0·619
Teaching	1654 (50·4)	1706 (53·0)	0·036	134 (48·4)	1044 (52·5)	316 (53·9)	212 (57·8)	0·107
Location			0·054					0·065
Midwest	611 (18·6)	627 (19·5)		56 (20·2)	399 (20·1)	100 (17·1)	72 (19·6)	
Northeast	407 (12·4)	453 (14·1)		33 (11·9)	279 (14·0)	81 (13·8)	60 (16·3)	
South	1789 (54·5)	1652 (51·4)		143 (51·6)	1006 (50·6)	334 (57·0)	169 (46·0)	
West	473 (14·4)	485 (15·1)		45 (16·2)	303 (15·2)	71 (12·1)	66 (18·0)	

Values in parentheses are percentages unless indicated otherwise;

* values are median (i.q.r.). CCI, Charlson Co‐morbidity Index; MI, myocardial infarction; COPD, chronic obstructive pulmonary disease.

† Pearson's χ^2^ test, except

‡ Mann–Whitney *U* test.

### Baseline characteristics by statin intensity levels

Among statin users, 277 (8·6 per cent) received low‐intensity statins, 1987 (61·8 per cent) moderate‐intensity statins, 586 (18·2 per cent) high‐intensity statins, and 367 (11·4 per cent) received supratherapeutic statins (*Table* 1). There was no difference in sex, insurance status, CCI score, admission type, perioperative beta‐blocker use, smoking status, and history of congestive heart failure, hypertension, chronic kidney disease, end‐stage renal disease, COPD and arrhythmia between the four statin intensity groups. Furthermore, hospital characteristics were comparable between the statin intensity groups. Patients who received high‐intensity statins were more likely to have a history of MI (22·5 *versus* 11·2 per cent) and coronary artery disease (62·5 *versus* 43·3 per cent) than those on low‐intensity statin therapy.

### In‐hospital postoperative acute myocardial infarction and major adverse events

MAE occurred in 2847 patients (43·8 per cent) undergoing open AAA repair (*Table* 2). The most common complications were respiratory (21·5 per cent), renal (20·1 per cent), cardiac (14·3 per cent) and infectious (12·5 per cent), whereas only 159 patients (2·4 per cent) developed MI. There was no difference in the crude risk of developing the composite MAE, respiratory, renal, haemorrhagic, infectious and neurological complications between statin users and non‐users. However, the crude risk of postoperative MI and the overall rate of cardiac complications were higher for statin users compared with non‐users. On the contrary, the crude risk of postoperative gastrointestinal complication was lower for statin users than non‐users. Among statin users, the crude risk of developing postoperative MAE was higher among patients who received a supratherapeutic dose than in patients taking high‐, moderate‐ or low‐intensity statins (*Table* 2).

**Table 2:** Crude postoperative outcomes by statin use and statin intensity

	Whole cohort			Statin users				
	No statin	Statin		Low	Moderate	High	Supratherapeutic	
	(*n* = 3280)	(*n* = 3217)	*P* *	(*n* = 277)	(*n* = 1987)	(*n* = 586)	(*n* = 367)	*P* *
Myocardial infarction	57 (1·7)	102 (3·2)	< 0·001	7 (2·5)	54 (2·7)	27 (4·6)	14 (3·8)	0·105†
Major adverse events	1429 (43·6)	1418 (44·1)	0·678	120 (43·3)	849 (42·7)	263 (44·9)	186 (50·7)	0·042
Respiratory failure	724 (22·1)	674 (21·0)	0·271	55 (19·9)	398 (20·0)	126 (21·5)	95 (25·9)	0·081
Cardiac	411 (12·5)	521 (16·2)	< 0·001	51 (18·4)	311 (15·7)	97 (16·6)	62 (16·9)	0·654
Gastrointestinal	140 (4·3)	73 (2·3)	< 0·001	6 (2·2)	43 (2·2)	14 (2·4)	10 (2·7)	0·888†
Haemorrhage	153 (4·7)	122 (3·8)	0·081	7 (2·5)	67 (3·4)	30 (5·1)	18 (4·9)	0·102†
Infectious	433 (13·2)	377 (11·7)	0·071	30 (10·8)	218 (11·0)	72 (12·3)	57 (15·5)	0·085
Neurological	18 (0·5)	28 (0·9)	0·122	2 (0·7)	17 (0·9)	6 (1·0)	3 (0·8)	0·123†
Renal failure	643 (19·6)	660 (20·5)	0·358	56 (20·2)	394 (19·8)	117 (20·0)	93 (25·3)	0·115
Death from any cause	206 (6·3)	83 (2·6)	< 0·001	9 (3·2)	42 (2·1)	20 (3·4)	12 (3·3)	0·168
Death following myocardial infarction	20 (35)	11 (10·8)	< 0·001	1 (14)	6 (11)	3 (11)	1 (7)	1·000†
Death following major adverse event	182 (12·7)	79 (5·6)	< 0·001	8 (6·7)	40 (4·7)	19 (7·2)	12 (6·5)	0·318

Values in parentheses are percentages.

* Pearson's χ^2^ test, except

† Fisher's exact test.

In the adjusted analysis, the odds of developing postoperative MAE were lower for moderate‐intensity statin users compared with those who did not take statins (odds ratio (OR) 0·86, 95 per cent c.i. 0·76 to 0·96); however, there was no difference in the odds of developing MAE for overall statin use (*versus* no statins), and for low, high and supratherapeutic statin intensity *versus* no statins (*Table* 3).

**Table 3:** Results of unadjusted and adjusted logistic regression analyses of mortality and major adverse events, by statin use and statin intensity

	Unadjusted		Adjusted*	
	Odds ratio	*P*	Odds ratio	*P*
Mortality*				
No statin	1·00 (reference)		1·00 (reference)	
Statin use	0·40 (0·30, 0·51)	< 0·001	0·41 (0·31, 0·54)	< 0·001
Statin intensity				
Low	0·50 (0·25, 0·99)	0·046	0·49 (0·24, 0·97)	0·041
Moderate	0·32 (0·23, 0·45)	< 0·001	0·34 (0·24, 0·47)	< 0·001
High	0·53 (0·33, 0·84)	0·007	0·56 (0·34, 0·90)	0·017
Supratherapeutic	0·50 (0·28, 0·91)	0·024	0·52 (0·28, 0·95)	0·035
Major adverse event†				
No statin	1·00 (reference)		1·00 (reference)	
Statin use	1·02 (0·93, 1·13)	0·678	0·90 (0·81, 1·00)	0·058
Statin intensity				
Low	0·99 (0·77, 1·27)	0·937	0·85 (0·66, 1·11)	0·232
Moderate	0·97 (0·86, 1·08)	0·551	0·86 (0·76, 0·96)	0·011
High	1·05 (0·88, 1·26)	0·555	0·94 (0·78, 1·14)	0·519
Supratherapeutic	1·33 (1·07, 1·65)	0·009	1·19 (0·95, 1·50)	0·132

Values in parentheses are 95 per cent confidence intervals.

* The logistic regression model for mortality was adjusted for patient age and sex, beta‐blocker use, chronic kidney disease, coronary artery disease, congestive heart failure, chronic obstructive pulmonary disease and admission status (elective, urgency or emergency).

† The model for major adverse events was adjusted for patient age, sex, race, primary payer, beta‐blocker use, chronic kidney disease, coronary artery disease, congestive heart failure, chronic obstructive pulmonary disease, history of acute myocardial infarction, admission status (elective, urgent or emergency), teaching and urban hospital status, and physician volume. In a *post hoc* analysis, using low‐intensity statins as a reference group, moderate, high and supratherapeutic statin intensity did not have a lower odds of death or major adverse events compared with low intensity.

### In‐hospital postoperative mortality and failure to rescue

The overall crude risk of postoperative death was lower for statin users than non‐users (*Table* 2). Similarly, the risk of death among patients who developed MI or MAE was lower for statin users. There were no differences between statin intensities in the risk of death from any cause, death following MI, and death following MAE.

In the adjusted analysis, patients who received a statin had lower odds of postoperative death compared with those who did not (OR 0·41, 0·31 to 0·54; *P* < 0·001) (*Table* 3). Moreover, moderate‐intensity statins had the lowest adjusted odds of mortality compared with no statin (OR 0·34, 0·24 to 0·47; *P* < 0·001). Of note, there was no difference in the adjusted odds of postoperative mortality between statin intensity levels.

## Discussion

In this study, statin use was associated with a 60 per cent reduction in the odds of death in hospital following open AAA repair. Although statin users were almost twice as likely to develop postoperative MI and other cardiac complications, among patients who developed MI, statin users had a lower risk of death than non‐users (10·8 *versus* 35·0 per cent). In patients who developed MAE, the risk of death was 55·9 per cent lower for statin users than non‐users. This indicates a rescue phenomenon associated with statin use.

There is inconsistent evidence in the literature describing the effect of statins on in‐hospital outcomes after vascular surgery. In a study by De Martino and colleagues^20^, the risk of in‐hospital death was the same for patients receiving a statin or a statin plus antiplatelet *versus* no statin among patients who underwent suprainguinal/infrainguinal bypass or open AAA repair. Similarly, in an analysis of 4721 Medicare patients, Galiñanes and co‐workers^12^ reported no significant difference in the risk of postoperative in‐hospital mortality for statin users *versus* non‐users after open AAA repair (5·1 *versus* 5·4 per cent; *P* = 0·66). Neither study included an adjusted analysis specifically among patients who underwent open repair but rather reported the crude risks of death. In contrast, a study^21^ of 997 patients undergoing vascular surgery (carotid endarterectomy, aortic repair and lower extremity revascularization) found statin therapy to be associated with 48 per cent lower odds of in‐hospital complications (death, MI, congestive heart failure and ventricular tachyarrhythmia) compared with no statin. The beneficial effects of statins on long‐term outcomes following open aneurysm repair have been demonstrated in multiple observational studies^4^. However, there have been no clinical trials assessing the association between statin therapy and postoperative outcomes following open AAA repair specifically. Even after vascular surgery in general, a recent Cochrane systematic review^15^ concluded that evidence is currently lacking with regard to the short‐term effects of statins on postoperative outcomes.

The present study investigated postoperative outcomes associated with different intensities of statin therapy specifically following open AAA repair. Moderate‐intensity statin therapy was associated with the greatest decrease in the odds of death (66 per cent lower odds of in‐hospital death compared with no statin therapy). Interestingly, there was no statistical difference in the adjusted odds of in‐hospital mortality between low, moderate, high and supratherapeutic statin intensity. Furthermore, moderate‐intensity statin therapy was the only intensity level to be associated with a lower risk of MAE compared with no statin. This is an important finding because of the growing advocacy for the use of intensive statin therapy for the purpose of lowering morbidity and mortality following vascular procedures, despite lack of evidence to support this practice. Most clinical trials comparing outcomes between different levels of statin intensity were performed in highly selected non‐surgical cohorts, excluding patients with diabetes, coronary artery disease or chronic kidney disease^22^, ^23^, ^24^, ^25^, ^26^, ^27^. How the findings from these clinical trials translate into real‐world improvement in outcomes is still a matter of debate. In a retrospective analysis of 15 729 Medicare patients (aged at least 65 years) with coronary artery disease, O'Brien and colleagues^28^ found no difference between high‐intensity *versus* low‐moderate‐intensity statin therapy with regard to 1‐ and 3‐year all‐cause mortality, major adverse cardiac events (MACE) and all‐cause readmission. Of note, more than 75 per cent of patients included in the present study were older than 65 years. Moreover, in an analysis of more than 7000 patients placed on statin therapy, Ross and co‐workers^29^ noted that the incidence of MACE was not determined by the statin intensity alone, but rather by achieving the target low‐density lipoprotein (LDL) level, and recommended investigating LDL titration strategies.

The present findings should be interpreted in light of multiple limitations, one of which concerns the administration pattern of statins. It was not possible to quantify the timing and duration of statin use before admission to hospital, which would have allowed identification of patients who used statins on a chronic basis and those who received statins prophylactically (within 30 days of admission). It is likely that this study is more representative of the effect of statins among chronic users, a notion supported by the observations of Patorno *et al*.^30^ in an analysis of more than half a million patients undergoing elective non‐cardiac procedures. They found that 22·5 per cent of patients received statins in the 6 months before non‐cardiac surgery, whereas only 1·2 per cent started statin therapy within 30 days before surgery. Selection bias is inherent in the retrospective design of this study; it was not possible to determine why some patients received low‐, moderate‐ or high‐intensity statins. In general, as described in the current ACC/AHA guidelines^19^, high‐intensity statin therapy is mainly recommended for patients aged between 40 and 75 years who are at high risk of atherosclerotic cardiovascular diseases, whereas moderate‐intensity statin therapy is recommended for patients older than 75 years. It is unlikely, therefore, that older patients with worse co‐morbidities would be more likely to receive high‐intensity statin therapy than younger patients, so the risk of selection bias is minimal. More than 10 per cent of patients who were on statin therapy received a supratherapeutic dose (based on the ACC/AHA guidelines^19^); the reason for this is unclear. This study is also limited by the lack of laboratory results, including serum LDL levels. It has been shown in a meta‐analysis^31^ of patients with coronary artery disease that there is a dose‐dependent relationship between reduction in MACE and serum LDL levels. Adjusting for this variable in logistic regression might affect the relationship between each intensity of statin therapy and postoperative outcomes. Although the present results did not find better outcomes associated with higher intensity of statins in this hospital setting, high and supratherapeutic intensity of statin therapy might prove beneficial to long‐term outcomes. Finally, although adjustment was made for the available measured confounders, without conducting an RCT with proper concealed treatment allocation, unmeasured confounders cannot be balanced between treatment groups. All findings in this study, therefore, represent measures of association rather than causation.

This large study has demonstrated that statin use reduces in‐hospital mortality following open AAA repair, with no evidence to suggest an additive beneficial effect associated with higher intensities of statin therapy in the hospital setting. This study emphasizes the need for optimum preoperative statin therapy before a planned open AAA repair.

## Disclosure

The authors declare no conflict of interest.

## Supporting information


**Table S1.** ICD‐9‐CM codes for Postoperative Major Adverse Events
**Table S2.** International Classification of Diseases – Ninth Revision – Clinical Modification (ICD‐9‐CM) Diagnosis Code for Comorbidity
**Table S3.** Total Count and Range (Minimum – Maximum) of Average Daily Statin Dose in Milligrams by Statin Type and Intensity
