# Supplementary material for: Statin intensity and postoperative mortality following open repair of intact abdominal aortic aneurysm
Source: BJS Open. 2018 Sep 6;2(6):411–8. doi: 10.1002/bjs5.94 (PMC6254010; doi:10.1002/bjs5.94)
Supplement: Supplementary file 1 — Table S1. ICD‐9‐CM codes for Postoperative Major Adverse Events Table S2. International Classification of Diseases – Ninth Revision – Clinical Modification (ICD‐9‐CM) Diagnosis Code for Comorbidity Table S3. Total Count and Range (Minimum – Maximum) of Average Daily Statin Dose in Milligrams by Statin Type and Intensity [file BJS5-2-411-s001.docx]

**BJS5_94**

**Statin intensity and postoperative mortality following open repair of intact abdominal aortic aneurysm**

H. N. Alshaikh, F. Bohsali, F. Gani, B. Nejim and M. Malas

**Table S1** ICD-9-CM codes for Postoperative Major Adverse Events

| ICD-9-CM code (Diag. or Proc.) | ICD-9-CM Code Description |
| --- | --- |
| Respiratory Failure | |
| 96.7x | Other continuous invasive mechanical ventilation |
| 93.9x | Respiratory therapy |
| 518.5 | Pulmonary insufficiency following trauma and surgery |
| 31.1 | Temporary tracheostomy |
| 31.2 | Permanent tracheostomy |
| Cardiac Complications | |
| 37.62 | Insertion of non-implantable heart assist system |
| 37.66 | Insertion of implantable heart assist system |
| 37.68 | Insertion of percutaneous external heart assist device |
| 97.44 | Non-operative removal of heart assist system |
| 427.0 | Paroxysmal supraventricular tachycardia |
| 427.1 | Paroxysmal ventricular tachycardia |
| 427.3x | atrial fib and flutter |
| 427.4x | Ventricular fibrillation and flutter |
| 37.21 | Right heart cardiac catheterization |
| 37.22 | Left heart cardiac catheterization |
| 37.23 | Combined right and left heart cardiac catheterization |
| 37.94 | ICD insertion |
| 99.61 | Atrial cardioversion |
| 99.62 | Other electric counter shock of heart |
| Major GI Dysfunction | |
| 574.0x | Calculus of gallbladder with acute cholecystitis |
| 574.3x | Calculus of bile duct with acute cholecystitis |
| 575.0 | Acute cholecystitis |
| 557.0 | Acute vascular insufficiency of intestine |
| 557.9 | Unspecified vascular insufficiency of intestine |
| 578.xx | GI bleed |
| 531.0x | Acute gastric ulcer with hemorrhage |
| 531.2x | Acute gastric ulcer with hemorrhage and perforation |
| 532.0x | Acute duodenal ulcer with hemorrhage |
| 532.2x | Acute duodenal ulcer with hemorrhage and perforation |
| 533.0x | Acute peptic ulcer with hemorrhage |
| 533.2x | Acute peptic ulcer with hemorrhage and perforation |
| 530.12 | Acute esophagitis |
| 535.xx | Gastritis and duodenitis |
| Hemorrhagic Dysfunction | |
| 998.11 | Hemorrhage complicating surgical procedure |
| 998.12 | Hematoma complicating surgical procedure |
| Infectious Complications | |
| 038.xx | Septicemia |
| 790.7 | Bacteremia |
| 041.xx | Bacterial infections unspecified site |
| 997.31 | Ventilator associated pneumonia |
| 481 | Pneumococcal pneumonia [Streptococcus pneumoniae pneumonia] |
| 482.41 | Methicillin susceptible pneumonia due to Staphylococcus aureus |
| 482.8x | Pneumonia due to other specified bacteria |
| 484.xx | Pneumonia in infectious diseases classified elsewhere |
| 486 | Pneumonia, organism unspecified |
| 510.xx | Empyema |
| 567.22 | Peritoneal abscess |
| 567.29 | Other suppurative peritonitis |
| 569.5x | Abscess of intestine |
| 996.xx | Complications peculiar to certain specified procedures |
| 519.2 | Mediastinitis |
| 995.91 | Sepsis |
| 995.92 | Severe sepsis |
| 998.3x | Disruption of wound/dehiscence |
| Renal Failure | |
| 584.xx | Acute renal failure |
| 586 | Renal failure, unspecified |
| 588.8 | Other specified disorders resulting from impaired renal function |
| 588.9 | Unspecified disorder resulting from impaired renal function |
| 639.3 | Renal failure, complication following abortion and ectopic and molar pregnancy |
| 39.95 | Dialysis |
| 38.95 | Venous catheter for renal dialysis |
| Neurologic Complication | |
| 431 | Intracerebral hemorrhage |
| 434.1x | Cerebral embolism |
| 434.9x | Cerebral artery occlusion, unspecified |
| 997.02 | Iatrogenic cerebrovascular infarction or hemorrhage, postoperative stroke |

Note: All codes presented in this table were adapted from a list of ICD-9-CM codes compiled by Shaw et al (2012).

Abbr. International Classification of Diseases – Ninth Revision – Clinical Modification (ICD-9-CM), Diagnosis (Diag.), Procedure (Proc.)

**Table S2** International Classification of Diseases – Ninth Revision – Clinical Modification (ICD-9-CM) Diagnosis Code for Comorbidity

| Comorbidity | ICD-9-CM Diagnosis Code |
| --- | --- |
| History of tobacco use | V15.82 |
| Current tobacco use | 305.1 |
| Hypertension | 401.x, 402.xx, 403.xx, 404.xx, 405.xx |
| History of MI | 412 |
| Coronary artery disease | 414.xx |
| Congestive heart failure | 428.xx |
| Chronic kidney disease | 585.1-585.5 |
| End stage renal disease | 585.6 |
| Chronic obstructive pulmonary disease | 490, 491.xx, 492.x, and 496 |
| Heart arrhythmia | 427.xx (except 427.5) |

**Table S3** Total Count and Range (Minimum – Maximum) of Average Daily Statin Dose in Milligrams by Statin Type and Intensity

| Type of statin | Statin Dose Intensity | | | | Total count |
| --- | --- | --- | --- | --- | --- |
|  | Low | Moderate | High | Supra-Therapeutic |  |
| Simvastatin | 5-18 | 20-40 | - | 42-140 | 1487 |
| Pravastatin | 10-27 | 40-80 | - | 85-160 | 448 |
| Rosuvastatin | - | 5-19 | 20-40 | 45-60 | 348 |
| Atorvastatin | - | 10-39 | 40-80 | 82-160 | 869 |
| Lovastatin | 10-27 | 40-40 | - | 44-80 | 65 |
| Total count | 277 | 1987 | 586 | 367 | 3217 |
